# Variables Reflecting the Mineralization of Bone Tissue From Fracturing Versus Nonfracturing Postmenopausal Nonosteoporotic Women

**DOI:** 10.1002/jbm4.10062

**Published:** 2018-06-20

**Authors:** Sébastien Rizzo, Delphine Farlay, Mohammed Akhter, Adele Boskey, Robert Recker, Joan Lappe, Georges Boivin

**Affiliations:** ^1^ INSERM, UMR 1033, Univ Lyon Université Claude Bernard Lyon 1 Lyon France; ^2^ Creighton University Osteoporosis Research Center, Omaha NE USA; ^3^ Hospital for Special Surgery New York NY USA

**Keywords:** FRACTURING POSTMENOPAUSAL WOMEN, NONFRACTURING POSTMENOPAUSAL WOMEN, DEGREE OF MINERALIZATION, HETEROGENEITY OF MINERALIZATION, DIGITIZED MICRORADIOGRAPHY

## Abstract

Women with equivalent areal bone mineral densities may show a different fracture incidence due to differences in bone intrinsic quality. Previously, Fourier transform infrared spectroscopic imaging (FTIRI) on the same iliac bone biopsies reported here, showed that the only significantly different variable was the carbonate/phosphate ratio, which was decreased in the fracturing group. Nanoindentation showed that fracturing bone was less mechanically heterogeneous than nonfracturing bone and could propagate damage (microcracks) more easily. The hypothesis is that fracturing women have reduced mineralization of bone tissue compared to nonfracturing women. Transiliac bone biopsies were collected from fracturing (*n* = 60, 62.5 ± 7.4 years old) and nonfracturing (*n* = 60, 62.3 ± 7.3 years old) postmenopausal women, to assess the mineralization of bone tissue using digitized microradiography. The degree of mineralization of bone (DMB, g/cm^3^) and the heterogeneity index (HI, g/cm^3^) of the DMB were calculated for cancellous (canc), cortical (cort) and total bone. Results were compared to variables from nanoindentation, FTIRI, and histomorphometry. DMB and HI were not significantly different between fracturing and nonfracturing groups. In the nonfracturing group, cort and canc HI were weakly negatively associated with cort and canc DMB (*r*′ = −0.388, *p *< 0.003; *r*′ = −0.532, *p *< 0.0001, respectively). In the fracturing group, DMB and HI were negatively correlated only in canc (*r*′ = −0.295, *p* = 0.024). DMB and HI were not associated with nanoindentation variables. Cort and canc DMB were positively associated with mineral‐to‐matrix ratio measured by FTIRI (ratio between mineral and organic matrix representing the relative mineralization of the collagen matrix), and negatively associated with carbonate/phosphate ratio. None of the DMB variables were strongly associated with any of the histomorphometric variables. In conclusion, bone mineralization was not significantly different between fracturing and nonfracturing postmenopausal women, suggesting that bone fragility could be partly due to other variables, such as changes in hydration of bone matrix or an increase of non‐enzymatic crosslinks in bone collagen. © 2018 The Authors. *JBMR Plus* published by Wiley Periodicals, Inc. on behalf of American Society for Bone and Mineral Research.

## Introduction

The osteoporotic (low‐trauma) fracture burden is a major public health problem. Up to 60% of women and 20% of men over 50 years of age are at risk of osteoporotic fracture, and the population over 50 years of age is increasing.^1^ However, women with equivalent areal bone mineral densities (aBMDs) may show a different fracture incidence due to differences in bone intrinsic quality. “Bone quality” refers to variables of bone matrix such as bone mineralization, mineral crystal properties, and collagen structure and characteristics.^2^ Bone is a living material having a hierarchical structure.^3^ Bone tissue is composed of a mineral phase contained in an organic matrix, the latter mainly consisting of type I collagen fibrils.^4^ Bone mineral is also a reservoir of ions that can be stored or released to maintain phosphocalcic equilibrium. The term “mineralization” involves not only the initial deposition of mineral in organic matrix but also its maturation until the upper mineral density in a given volume of matrix is reached.^2^ The latter includes an increase in number, size, and perfection of crystals. Independent of bone mass and its distribution in space, the mineralization and the “quality” of the mineral play a crucial role in the elastic, plastic, and viscoelastic properties defining the mechanical behavior of bones.^5^, ^6^ At the tissue level, bone is composed of bone structural units (BSUs), the osteons in cortical bone and bone packets in trabecular bone. In adults, these BSUs correspond to the production of bone tissue as result of remodeling cycles. The first step in mineralization at each remodeling site starts 5 to 10 days after the initial deposition of organic matrix by osteoblasts during bone formation.^7^ Because bone remodeling occurs asynchronously in various regions of a given bone, it results in a heterogeneous distribution of mineralization throughout bone tissue. The degree of mineralization of each BSU is thus dependent on the time since its deposition.^8^, ^9^ The degree of mineralization and its distribution are tightly related to the activation frequencies of bone remodeling cycles, which can be influenced by age, hormonal status (such as low estrogen levels after menopause), pathologies, or therapy.^2^, ^7^, ^9^

In a large biopsy study, including 120 iliac bone samples from otherwise healthy postmenopausal women with and without fragility fractures, paired by age and aBMD, the fracture status was discriminated by variables reflecting bone quality at the tissue level.^10^ Indeed, in this study, using conditional logistic regression analyses, women experiencing a fracture had a lower carbonate‐to‐phosphate ratio in both cortical and cancellous bone, and a higher heterogeneity of collagen maturity for cancellous bone compared to their nonfracturing pairs. Some of the fracture risk associated with aging may be related to age‐related changes in crystallinity, but collagen maturity is likely a marker for age‐related changes in bone rather than directly associated with excess fracture risk. In the same iliac bone samples, nanoindentation suggested that the subjects with fracturing bone had less heterogeneous material properties (cortical hardness, modulus storage modulus, and trabecular hardness) compared to the nonfracturing ones, and therefore, were more prone to fracture. The higher variation in loss modulus for the fracturing bone suggested there was existing damage in bone tissue that could propagate more easily in the relatively homogenous material, and therefore, may have a role in the increased risk of fracture.^11^

Our hypothesis is that postmenopausal, nonosteoporotic, but osteopenic, women who have sustained fractures have compromised bone quality as indicated by measuring variables reflecting the mineralization of bone tissue, compared to bone from postmenopausal, nonosteoporotic, but similarly osteopenic women who have not fractured.

## Subjects and Methods

### Participants

One hundred and twenty postmenopausal women were recruited for a study of bone quality. The fracture group (cases) included 60 postmenopausal women with osteopenic BMD values (*T*‐scores between −1.0 and −2.5 for either the hip or spine), who were between ages 45 and 80 years, had a fracture during the previous 5 years from low trauma, but were otherwise healthy. “Low‐trauma fracture” was defined as any fracture caused by trauma equal to, or less than, a fall to the floor from a standing height, excluding fractures of the digits, face, or skull. The low‐trauma fractures in the cases occurred as follows: wrist (*n* = 20), ankle (*n* = 16), humerus (*n* = 7), patella (*n* = 4), shoulder (*n* = 3), elbow (*n* = 2), hip fracture (*n* = 2), and other locations with one fracture (fibula, foot, knee, lower leg, pelvis, and wrist and elbow combined) (*n* = 6). At least one vertebral fracture was reported in 23 individuals. In contrast, six of the controls had a history of fracture, none were low‐trauma, all were from motor vehicle accidents, or similar levels of trauma. None of the women were on any antiresorptive (bisphosphonate, calcitonin, estrogen, etc.) or bone‐forming agents (PTH). The control group included 60 postmenopausal women who had no prevalent osteoporotic fractures (by history or spine X‐ray) on entry into study. As in the fracturing group, all of the control subjects were between ages 45 and 80 years, had *T*‐scores between −1.0 and −2.5 for either the hip or spine, and were otherwise healthy. Each fracturing subject was matched with a control subject who was similar in age and within 10% of BMD. The matching was performed immediately after the successful enrollment of each fracturing subject. None of the subjects had significant reduction in activities of daily living (ADL). History of systemic, metabolic, or endocrine diseases, diabetes, or chronic kidney diseases, were never reported in any of the subjects from the present study. Serum 25OHD was measured in all subjects. There was no significant difference between fracturing cases and controls in serum 25OHD. The participants have given informed consents and the research was conducted in accordance with the principles laid down in the Declaration of Helsinki. The project was reviewed and approved by the institutional review board.

Two transiliac bone biopsies were collected from each fracturing subject (*n* = 60, 62.5 ± 7.4 years old, taken within 5 months after a fracture), and each nonfracturing control (*n* = 60, 62.3 ± 7.3 years old). The two biopsies were taken from opposite sides of the pelvis in each case. Transiliac bone biopsies were done using a Meunier's trephine having a 7.5‐mm inner diameter. Bone biopsies were performed 2 cm inferior and posterior to the anterior‐superior iliac spine. Fragility fractures (low‐trauma) were defined as fractures occurring from trauma less than or equal to a fall to the floor from a standing height, excluding fractures of digits, face or skull, and were located at the wrist, ankle, patella, shoulder, humerus, and other locations, with one or two fractures from each location. Each fracturing case was matched by age and aBMD with a nonfracturing control recruited at the time each fracturing case was recruited. All were postmenopausal nonosteoporotic women with BMD *T*‐scores between −1.5 and −2.5. We assessed the mineralization of bone tissue of both groups using a digitized microradiography device.^12^ One biopsy specimen (7.5 mm in diameter) from each subject was fixed in alcohol, dehydrated, and embedded in polymethylmethacrylate (PMMA) without prior decalcification, and had been used for previous analyses (histomorphometry, Fourier transform infrared spectroscopic imaging [FTIRI]). The other biopsy specimen from each subject was embedded in epoxy resin for the nanoindentation study, and the studies described here.^11^ For each participant, one specimen was embedded in epoxy resin (EpoThin, Buehler, IL, USA), then ground and polished, and used only for the nanoindentation studies and the studies described herein. Each bone biopsy was carefully cleaned of bone marrow using a soft jet of ionic water. After removing the residual water via centrifuge at 3000 rpm for 30 s while retaining the water located in the bone tissue (the tissue remained in the hydrated state), the biopsy was embedded in low viscosity EpoThin embedding material. The approximate centrifuge was at 3000 rpm (revolution per minute). The “Relative Centrifugal Force” (RCF) was 1286 × g. EpoThin epoxy uses resin and hardener (1:3 ratio) similar to our previous study.^11^ The embedding material flows into the small crevices, cracks, holes, etc., and hardens within 24 hours without penetrating or diffusing into bone tissue itself. The EpoThin embedding material provides mechanical support to the bone tissue being tested under compressive forces. A section of approximately 150 μm in thickness was cut using a precision diamond wire saw (Escil, Chassieu, France). The sections were further thinned to 100 ± 1 μm by manually grinding them between a frosted glass plate and a frosted glass slide using silicon carbide powder (Escil, Chassieu, France). The sections were polished using 1 μm alumina suspension (Escil, Chassieu, France) and cleaned in an ultrasonic device (Elma, Singen, Germany). The thickness was measured with a precision micrometric thickness comparator (precision of 1 μm; Compac, Geneva, Switzerland).

### Quantitative digitized microradiography

**Figure 1:**
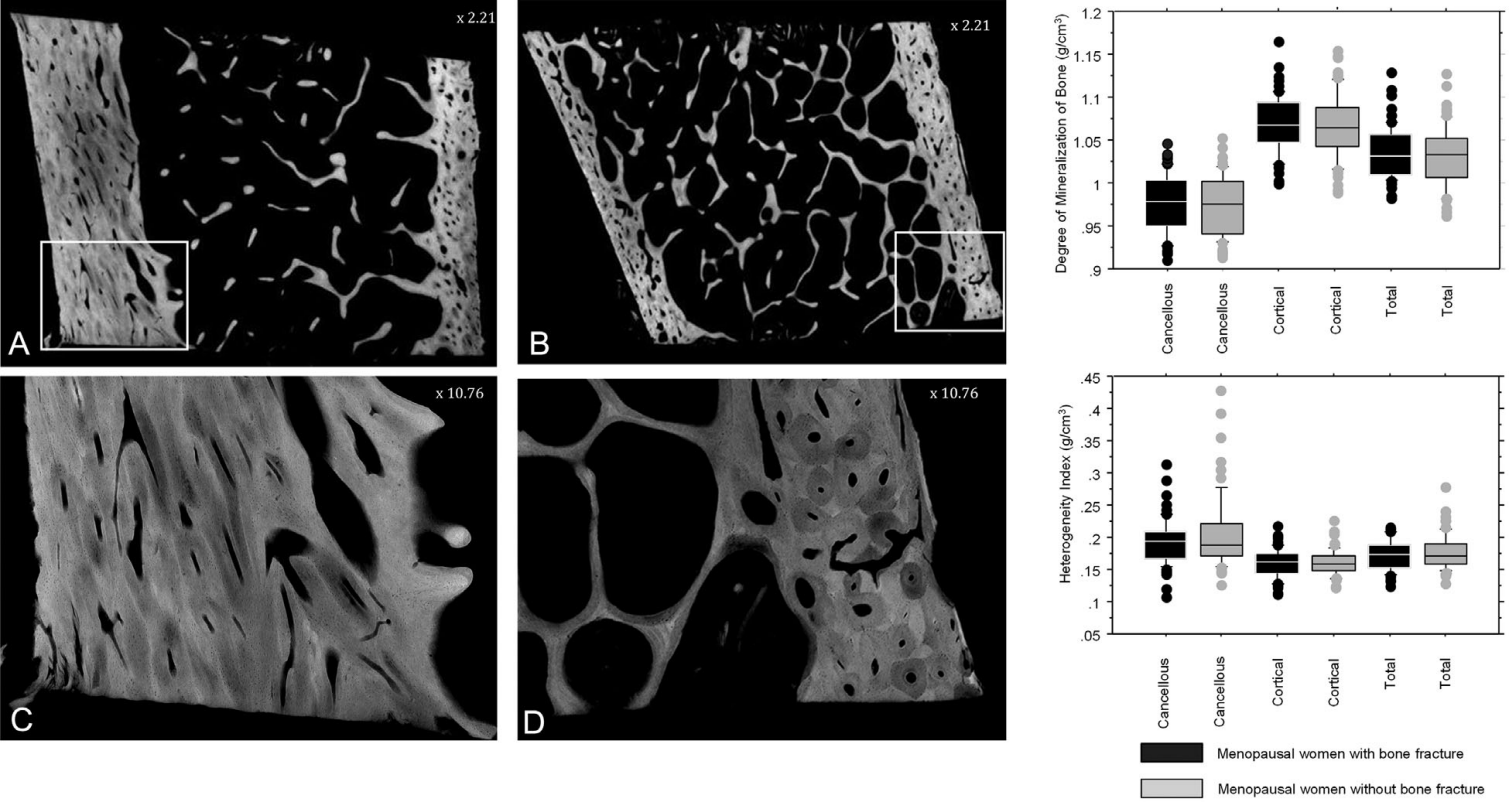
Digitized microradiographs of sections of iliac bone biopsies from fracturing (*A*, *C*) versus nonfracturing (*B*, *D*) postmenopausal nonosteoporotic women. *C* and *D* are identified areas from *A* and *B* and observed at higher magnification. Box plots on the right show that the degree of mineralization of bone and the heterogeneity index of iliac bone biopsies are not significantly different in fracturing nonosteoporotic postmenopausal women versus nonfracturing, nonosteoporotic postmenopausal women whatever the bone compartment assessed.

The digitizing device was developed in collaboration with Photonic Science (St Etienne de St Geoirs, France). It is composed of a Microfocus Hammamatsu X‐ray system, L9421‐02, with a power maximum of 8 W and a focal spot size of 5 µm in diameter, a copper anode (K*α* radiation energy of 8.05 KeV), a nickel filter, and a 150‐µm‐thick Beryllium window. The exposure parameters were as follows: high voltage: 40 kV, current: 50 µA, and power: 2 W. The detector was a Photonic science CCD camera FDI VHR 11 M with an active area of 36 × 24 mm (4008 × 2671 pixels; pixel size: 9 µm). The digital system dynamic range is 12 bits (ie, 4096 values) allowing precise gray level information to be obtained (Fig. 1A–D).^12^

For the quantitative evaluation of X‐ray absorption by bone tissue, an aluminum reference system with a known absorption coefficient was exposed to the beam prior to exposure to the bone section (aluminum was selected because it has a low atomic number close to that of hydroxyapatite). The reference system was constructed in the shape of a rectangular staircase with steps consisting of one to eight layers of ultrapure aluminum (99.5%; Strems Chemical Ltd, Strasbourg, France).^8^ Gray levels obtained at pixel levels of the entire bone section were converted into the degree of mineralization of bone after plotting a calibration curve based on the values obtained from the aluminum standard. The range of gray level values was chosen in order to reduce the risk of having too little bone tissue, combined with lots of PMMA.

Finally, variables reflecting the secondary mineralization of bone, were assessed in a blinded fashion on digitized microradiographs using a code from the Matlab program and were expressed in g mineral/cm^3^ of bone (adjusted to the exact thickness of each part of the section). These variables are the degree of mineralization of bone (DMB) and the heterogeneity index (HI = full width at half maximum of the curve of distribution of the mineralization [the greater the value of HI, the greater the heterogeneity of mineralization]). These variables were given for each compartment of bone tissue: cortical, cancellous, and total bone (= cortical + cancellous). For cortical data, all the internal and external cortex were used to assess the mineralization variables.

For each bone compartment, the intraobserver reproducibility is ≤1.5% for DMB and ≤5.1% for HI, and the interobserver reproducibility is ≤0.6% for DMB and ≤3.2% for HI (unpublished data).

During analyses of the samples, the operator was blinded to the assignment of the samples. Variables reflecting mineralization of bone were compared to intrinsic strength properties measured using the nanoindentation method,^11^ to bone quality characteristics determined by FTIRI,^9^ and to bone histomorphometric analysis (Recker and colleagues, unpublished data). Nanoindentation testing was performed on both cortices. However, the anatomical direction/orientation of the cortical bone tissue within each biopsy was not certain. Therefore, the internal and external cortices could not be distinguished.^11^ The epoxy resin did not affect nanoindentation testing of bone tissue. The epoxy resin (EpoThin) was a quick‐setting plastic that hardened in a short time within the pores and crevices of bone without penetrating or diffusing into the bone tissue.^11^

### Statistical analysis

Microradiography, FTIRI, and nanoindentation were performed on adjacent slices of transiliac bone biopsies. Comparison between both groups (*n* = 60 in each group) was performed using a nonparametric Mann‐Whitney *U* test. Spearman correlations were used to test the relationships between different variables. Statistical significance required a *p* < 0.05. All statistical tests were performed with Statview v5.0.1 (SAS Institute, Inc., Cary, NC, USA).

## Results and Discussion

Variables reflecting mineralization of bone (DMB and HI) were not significantly different in fracturing nonosteoporotic postmenopausal women compared to nonfracturing ones, whatever the bone compartment (Fig. 1). In cortical, cancellous and total bone, respectively, mean ± SD DMB (g/cm^3^) were 1.064 ± 0.038, 0.974 ± 0.035, and 1.031 ± 0.036 in nonfracturing women versus 1.068 ± 0.034, 0.977 ± 0.033, and 1.034 ± 0.031 in fracturing women. In cortical, cancellous, and total bone, respectively, mean ± SD HI (g/cm^3^) were 0.159 ± 0.020, 0.205 ± 0.058, and 0.177 ± 0.027 in nonfracturing women versus 0.159 ± 0.023, 0.193 ± 0.036, and 0.172 ± 0.024 in fracturing women. In each group, DMB and HI were significantly different between cortical and cancellous bone (*p *< 0.0001); DMB was lower and HI higher in cancellous than in cortical bone, in agreement with the higher bone remodeling activity in cancellous bone than in cortical bone. Total bone DMB and HI were strongly associated with cortical data, due to the greater amount of cortical versus trabecular bone volume. In the nonfracturing group, cortical and cancellous HI were weakly negatively associated with cortical and cancellous DMB (*r*′ = −0.388, *p* = 0.0029; *r*′ = −0.531, *p *< 0.0001, respectively); ie, higher DMB values were associated with lower HI. In the fracturing group, DMB and HI were negatively correlated only in cancellous (*r*′ = −0.295, *p* = 0.024). In the nonfracturing group, negative correlations in both cortical and cancellous bone indicate that a high degree of mineralization is associated with a low heterogeneity of mineralization, thus there is a direct relationship between bone remodeling and mineralization. These correlations are not observed in fracturing‐group in cortical bone only, suggesting that the relationship between bone remodeling and mineralization is not so clear in this group, and that another factor might impact mineralization independently of bone remodeling. Variables reflecting mineralization of bone (DMB and HI) were not significantly correlated with serum 25OHD.

A decrease in hardness, independent of DMB, has already been shown and was associated with a decrease in mineral crystallinity.^13^ Indeed, the strength deficits were, in part, related to differences in crystallinity, irrespective of the mineral amount and maturity.

DMB and HI were not strongly associated with any of the nanoindentation variables.^11^ There were only significant (*p *≤ 0.05), but weak, negative correlations between cortical DMB and cortical hardness or cortical storage modulus. This can also be due to the fact that the measurement sites of DMB and nanoindentation were not strictly colocalized, because DMB was measured on the entire bone biopsy and nanoindentation at specific locations.

Cortical and cancellous DMB were positively associated with index of mineralization assessed by FTIRI,^10^ while DMB (measured by digitized microradiography) is the absolute value of mineralization, and mineral‐to‐matrix ratio (measured by FTIRI) is the content of mineral relative to organic matrix. Cortical and cancellous DMB were negatively associated with carbonate‐to‐phosphate ratio.^10^ Those results are in agreement with a previous study performed on ewes showing a decrease in carbonation with increase in mineralization.^7^ However, the evolution of carbonate‐to‐phosphate ratio with bone matrix maturity is conflicting in the literature, with some studies showing an increase^14^ or a decrease^15^ of the ratio with maturation. We have not assessed mineralization into and around osteocyte lacunae. In the microradiographs, no highly mineralized periosteocytic lacunae, and no micropetrosis were observed. We fully agree with the fact that reduced osteocyte number and the presence of microcracks may be also a cause of bone fragility. Osteocyte number and microcracks were not assessed in the present study.

Carbonate content affects solubility of bone mineral.^16^ Indeed, when CO_3_^2–^ are incorporated in bone mineral (substituted for PO_4_^3–^) a vacancy is created to maintain electrostatic equilibrium. However, the presence of vacancies increases solubility of bone mineral. The factors that affect the carbonate‐to‐phosphate ratio are unclear, but it has been suggested that disturbance in the acid‐base equilibrium in bone marrow can modulate the carbonate‐to‐phosphate ratio in renal disease when this equilibrium is impaired^17^ or in patients with metastatic prostate cancer.^18^

The conflicting results in the literature come from the fact that it is difficult to measure exactly the same type of carbonate (type A, type B, or labile) by different techniques (Raman or FTIR spectroscopy). By definition, labile carbonates are unstable, and fixation and embedding can modify this type of carbonate. Moreover, by Raman or FTIRI spectroscopy, the presence of overlapping bands (mineral vibration as HPO_4_ or organic contributions) can lead to some difficulty in extracting the pure contribution of each type of carbonate and lead to different results.

DMB and HI were not strongly associated with any of the histomorphometric variables. However, there were weak, but significant, correlations (*p *≤ 0.05); ie, cortical and cancellous DMB were positively correlated with mineral apposition rates. Regarding the age distribution, histomorphometry data have been published on 34 normal postmenopausal women and in perimenopausal and postmenopausal women.^19^, ^20^ The histomorphometry data in the current group of women are not significantly different from the data from the postmenopausal women of similar age and time since menopause in those two groups. Also, age‐related changes in those two groups of similar age were minimal and not significant.

This study has two main limitations. The first one is that iliac bone tissue may not reflect what happens in long bones, and therefore the lack of difference between fracturing and nonfracturing patients could be related to the site analyzed (greater loading versus lower loading of bone). In addition, the remodeling rate in trabecular bone is about 15%/year, but in cortical bone is about 3%/year.^21^, ^22^ The second is that bone biopsies were taken within 5 years after the low trauma fractures, and this could affect the results if bone intrinsic quality changed between time of fracture and bone biopsy.

In conclusion, variables reflecting bone mineralization are not significantly different between fracturing and nonfracturing postmenopausal women. This may suggest that fragility fracture may be driven by impairments in bone quality at levels of organization lower than the tissue scale. Bone fragility could be partly due to other variables such as changes in hydration of bone matrix or an increase in non‐enzymatic crosslinks in bone collagen. It is well established that the dehydration of bone causes brittleness by increasing stiffness. Several types of water (free, bound, presence in collagen or mineral) exist in bone and their respective roles on material properties are nicely described.^23^, ^24^ Recently, it has been demonstrated that water bound to the apatite surface helps to orient crystals during mineralization.^25^ Moreover, when collagen mineralizes a part of this water is replaced.^26^

The major role of osteocytes in bone mechanosensation and repairing microcracks is well documented. Recently, impaired mechanosensitivity of osteocytes associated with estrogen deficiency, has been shown to cause risk of osteoporotic bone fractures.^27^ Moreover, the decrease in osteocyte viability impairs microcrack repair, and thus the dissipation of energy during physiological loading would cause an excessive number of microcracks that weaken bone and lead to fracture.^28^, ^29^

## Disclosures

Sebastien Rizzo, Delphine Farlay, Mohammed Akhter, Adele Boskey, Robert Recker, Joan Lappe, and Georges Boivin state that they have no conflicts of interest.

## References

[1] World Health Organization. Prevention and management of osteoporosis. World Health Organization Technical Report Series. 2003;921:1–64. Available from: http://www.who.int/iris/handle/10665/42841. 15293701

[2] Bala Y , Farlay D , Boivin G. Bone mineralization: from tissue to crystal in normal and pathological contexts. Osteoporos Int. 2013;24:2153–66. 23229470 10.1007/s00198-012-2228-y

[3] Rho JY , Kuhn‐Spearing L , Zioupos P. Mechanical properties and the hierarchical structure of bone. Med Eng Phys. 1998;20:92–102. 9679227 10.1016/s1350-4533(98)00007-1

[4] Boskey AL. Bone mineral crystal size. Osteoporos Int. 2003;14 Suppl 5:16–21. 10.1007/s00198-003-1468-2 14504701

[5] Ferguson VL. Deformation partitioning provides insight into elastic, plastic, and viscous contributions to bone material behavior. J Mech Behav Biomed Mater. 2009;2:364–74. 19627843 10.1016/j.jmbbm.2009.01.004

[6] Bala Y , Depalle B , Douillard T , et al. Respective roles of organic and mineral components of human cortical bone matrix in micro‐ mechanical behavior: an instrumented study. Journal of Biomechanical Behavior of Biomedical Materials. 2011;4:1473–82. 10.1016/j.jmbbm.2011.05.017 21783157

[7] Bala Y , Farlay D , Delmas PD , Meunier PJ , Boivin G. Time sequence of secondary mineralization and microhardness in cortical and cancellous bone from ewes. Bone. 2010;46:1204–12. 19969115 10.1016/j.bone.2009.11.032

[8] Boivin G , Meunier PJ. Changes in bone remodeling rate influence the degree of mineralization of bone. Connect Tissue Res. 2002;43:535–7. 12489211 10.1080/03008200290000934

[9] Roschger P , Paschalis EP , Fratzl P , Klaushofer K. Bone mineralization density distribution in health and disease. Bone. 2008;42:456–66. 18096457 10.1016/j.bone.2007.10.021

[10] Boskey AL , Donnelly E , Boskey E , et al. Examining the relationships between bone tissue composition, compositional heterogeneity, and fragility fracture: a matched case‐controlled FTIRI study. J Bone Miner Res. 2016;31:1070–81. 26636271 10.1002/jbmr.2759 PMC4862946

[11] Vennin S , Desyatova A , Turner JA , et al. Intrinsic material property differences in bone tissue from patients suffering low‐trauma osteoporotic fractures, compared to matched non‐fracturing women. Bone. 2017;97:233–42. 28132909 10.1016/j.bone.2017.01.031 PMC5367951

[12] Montagner F , Kaftandjian V , Farlay D , Brau D , Boivin G , Follet H. Validation of a novel microradiography device for characterization of bone mineralization. J Xray Sci Technol. 2015;23:201–11. 25882731 10.3233/XST-150481

[13] Bala Y , Depalle B , Farlay D , et al. Bone micromechanical properties are compromised during long‐term alendronate therapy independently of mineralization. J Bone Miner Res. 2012;27:825–34. 22189833 10.1002/jbmr.1501

[14] Petra M , Anastassopoulou J , Theologis T , Theophanides T. Synchrotron micro‐FTIR spectroscopic evaluation of normal paediatric human bone. J Molec Struct. 2005;733:101–10.

[15] Ou‐Yang H , Paschalis EP , Mayo WE , Boskey AL , Mendelsohn R. Infrared microscopic imaging of bone: spatial distribution of CO_3_ ^(2−)^ . J Bone Miner Res. 2001;16:893–900. 11341334 10.1359/jbmr.2001.16.5.893

[16] LeGeros RZ. Apatites in biological systems. Progress in Crystal Growth and Characterization of Materials. 1981;4:1–45.

[17] Bacchetta J , Farlay D , Abelin‐Genevois K , Lebourg L , Cochat P , Boivin G. Bone impairment in oxalosis: an ultrastructural bone analysis. Bone. 2015;81:161–7. 26164477 10.1016/j.bone.2015.07.010

[18] Bi X , Sterling JA , Merkel AR , Perrien DS , Nyman JS , Mahadevan‐Jansen A. Prostate cancer metastases alter bone mineral and matrix composition independent of effects on bone architecture in mice—a quantitative study using microCT and Raman spectroscopy. Bone. 2013;56:454–60. 23867219 10.1016/j.bone.2013.07.006 PMC3799839

[19] Recker RR , Kimmel DB , Parfitt AM , Davies KM , Keshawarz N , Hinders S. Static and tetracycline‐based bone histomorphometric data from 34 normal postmenopausal females. J Bone Miner Res. 1988;3:133–44. 3213608 10.1002/jbmr.5650030203

[20] Recker RR , Kimmel DB , Lappe JM , Coble T. Iliac bone histomorphometry in normal peri‐ and postmenopausal women. J Bone Miner Res. 1990;5(S1):S118 (Abstract 180 at 12th Annual Meeting ASBMR, Atlanta, GA, USA, Aug. 28–31 1990).

[21] Frost HM. Tetracycline‐based histological analysis of bone remodeling. Calcif Tissue Res. 1969;3:211–37. 4894738 10.1007/BF02058664

[22] Frost HM. Dynamics of bone remodeling. Boston: Little, Brown & Co; 1964 p. 315–33.

[23] Granke M , Does MD , Nyman JS. The role of water compartments in the material properties of cortical bone. Calcif Tissue Int. 2015;97:292–307. 25783011 10.1007/s00223-015-9977-5 PMC4526331

[24] Nyman JS , Roy A , Shen X , Acuna RL , Tyler JH , Wang X. The influence of water removal on the strength and toughness of cortical bone. J Biomech. 2006;39:931–8. 16488231 10.1016/j.jbiomech.2005.01.012 PMC1941695

[25] Wang Y , Von Euw S , Fernandes FM , et al. Water‐mediated structuring of bone apatite. Nat Mater. 2013;12:1144–53. 24193662 10.1038/nmat3787

[26] Robinson RA. Physicochemical structure of bone. Clin Orthop Rel Res. 1975;263–315. 1192643

[27] Klein‐Nulend J , Van Oers RFM , Bakker AD , Bacabac RG. Bone cell mechano‐sensitivity, estrogen deficiency, and osteoporosis. J Biomech. 2015;48:855–65. 25582356 10.1016/j.jbiomech.2014.12.007

[28] Burr DB , Turner CH , Naick P , et al. Does microdamage accumulation affect the mechanical properties of bone? J Biomech. 1998;31:337–45. 9672087 10.1016/s0021-9290(98)00016-5

[29] Donahue SW , Galley SA. Microdamage in bone: implications for fracture, repair, remodeling and adaptation. Crit Rev Biomed Eng. 2006;34:215–71. 16930125 10.1615/critrevbiomedeng.v34.i3.20

